# Different loss of material in recurrent chromosome 20 interstitial deletions in Shwachman-Diamond syndrome and in myeloid neoplasms

**DOI:** 10.1186/1755-8166-6-56

**Published:** 2013-12-12

**Authors:** Roberto Valli, Barbara Pressato, Cristina Marletta, Lydia Mare, Giuseppe Montalbano, Francesco Lo Curto, Francesco Pasquali, Emanuela Maserati

**Affiliations:** 1 Dipartimento di Medicina Clinica e Sperimentale, Università dell’Insubria, Via J. H. Dunant, 5, I 21100 Varese, Italy

**Keywords:** Shwachman-Diamond syndrome, del(20)(q), Myeloid neoplams

## Abstract

**Background:**

An interstitial deletion of the long arms of chromosome 20, del(20)(q), is frequent in the bone marrow (BM) of patients with myelodysplastic syndromes (MDS), acute myeloid leukemia (AML), and myeloproliferative neoplasms (MPN), and it is recurrent in the BM of patients with Shwachman-Diamond syndrome (SDS), who have a 30-40% risk of developing MDS and AML.

**Results:**

We report the results obtained by microarray-based comparative genomic hybridization (a-CGH) in six patients with SDS, and we compare the loss of chromosome 20 material with one patient with MDS, and with data on 92 informative patients with MDS/AML/MPN and del(20)(q) collected from the literature.

**Conclusions:**

The chromosome material lost in MDS/AML/MPN is highly variable with no identifiable common deleted regions, whereas in SDS the loss is more uniform: in 3/6 patients it was almost identical, and the breakpoints that we defined are probably common to most patients from the literature. In some SDS patients less material may be lost, due to different distal breakpoints, but the proximal breakpoint is in the same region, always leading to the loss of the *EIF6* gene, an event which was related to a lower risk of MDS/AML in comparison with other patients.

## Background

Shwachman-Diamond syndrome (SDS) is an autosomal recessive disorder (Online Mendelian Inheritance in Man #260400) that is caused by mutations of the *SBDS* gene in at least 90% of cases [1]. An interstitial deletion of the long arms of chromosome 20, del(20)(q), is recurrent as an acquired abnormality in the bone marrow (BM) of patients with Shwachman-Diamond syndrome (SDS) [1], as well as in myelodysplastic syndromes (MDS), acute myeloid leukemia (AML), and myeloproliferative neoplasms (MPN) [2]. The fact that SDS patients have a risk of developing MDS/AML, evaluated as high as 30-40% [1,3], suggested that this clonal chromosome anomaly may be responsible of MDS/AML, although some evidence indicates that this specific anomaly in SDS is associated with a rather low risk [4]. We postulated that the low risk of MDS/AML is due to the loss of the *EIF6* gene, mapping in the deleted segment of chromosome 20: the function of the EIF6 protein is pivotal in ribosome biogenesis, and the gene/dosage effect consequent to the gene loss would facilitate ribosome formation, impaired in SDS by *SBDS* mutations [5].

In patients with MDS/AML/MPN and del(20)(q), some attempts have been made to establish the smallest common deleted region (CDR) by cytogenetic and molecular genetic methods [6,7]: the results were partially consistent and indicated a CDR of 250 Kb – 1.7 Mb within the chromosome band 20q12. The introduction of array methods, as microarray-based comparative genomic hybridization (a-CGH) and single nucleotide polymorphism arrays (SNP-array), was expected to define more precisely one or more CDR, possibly containing genes relevant for the pathogenesis of myeloid neoplasms. According to available literature this has not been the case: different conclusions were drawn, e.g., by Huh et al. [8] and Okada et al. [9].

We report here data obtained by means of a-CGH on six SDS patients carrying the del(20)(q): four of them were already partially described, and they are identified in our laboratory and in our previous reports with their Unique Patient Number (UPN) as 13, 14, 17, and 20. Two of them, UPN 13 and 14, acquired the del(20)(q) during the follow-up, after our previous report [10]. UPN 17 and UPN 20 showed also a clone in which the del(20)(q) was further rearranged, with a complex pattern including deletions of both the short and the long arms and tiny duplications of the long arms [11]. The other two, unreported, SDS patient are: UPN 65, a 13-year-old male, with a diagnosis of SDS made at one year of age, homozygous for the mutation 258 + 2 T > C of the *SBDS* gene; UPN 68, a 19-year-old male, with diagnosis of SDS at 10 years of age, with the mutations of the *SBDS* gene 258 + 2 T > C/183-184TA > CT + 258 + 2 T > C. One patient with MDS was compared with the SDS patients: he was a 5-year-old boy with a diagnosis of refractory cytopenia with unilinear dysplasia (anaemia) (RCUD) in whom an acquired del(20)(q) was found in BM, and was defined as interstitial by a-CGH.

In this article we analyse the results obtained by a-CGH in our six SDS patients, and we compare the loss of chromosome 20 material with one patient affected by MDS and with 102 patients with MDS/AML/MPN collected from the literature, all investigated by a-CGH or SNP-array.

## Results

Table 1 summarizes all relevant cytogenetic data obtained on BM cells at the time of a-CGH. In particular, the proportion of cells bearing the del(20)(q) is evaluable from chromosome analyses and from the results of fluorescent in situ hybridization (FISH), also on nuclei, performed with informative probes. In all cases, the percentage of abnormal cells was above the limit of detectability in a-CGH assay [12].

**Table 1:** Cytogenetic and a-CGH results on BM cells of SDS and MDS patients

**Patient**	**Karyotype**	**FISH on mitoses**^**a**^	**FISH on nuclei**^**a**^	**a-CGH**^**b**^**: 20q loss bp**^**c **^**position (% abnormal cells)**^**d**^
UPN 13	46,XY,del(20)(q11.21q13.32)[2]/46,XY[2]	9/21 (42.8%) 1 signal	184/366 (50.3%) 1 signal	30 876 455 – 57 739 561 bp (55%)
UPN 14	46,XY[49]	30/170 (17.6%) 1 signal	68/470 (14,5%) 1 signal	31 163 090 – 35 309 353 bp (18.2%)
UPN 17	46,XY,del(20)(q11.21q13.31)[5]/46,XY,der(20)del(20)(p)del(20)(q)dup(20)(q)[20]/46,XY[6]	na^e^	na	31 205 853 – 55 894 832 bp (46.9%)
UPN 20	46,XY,del(20)(q11.21q13.32)[18]/46,XY,der(20)del(20)(p)del(20)(q)dup(20)(q)[6]/46,XY[2]	9/10 (90%) 1 signal	na	31 294 381 – 57 252 304 bp (66.5%)
UPN 65	46,XY,del(20)(q11.21q13.13)[6]/46,XY[13]	7/17 (41%) 1 signal	191/619 (30.8%) 1 signal	30 157 286 – 49 497 910 bp (43%)
UPN 68	46,XY,del(20)(q11.21q13.13)[2]/46,XY[14]	na	82/612 (13.4%) 1 signal	31 262 228 – 43 141 564 bp 45 244 728 – 47 373 129 bp (15.9%)
MDS pt.^f^	46,XY,del(20)(q11.23q13.32)[7]/46,XY[4]	59/70 (84.3%) 1 signal	450/581 (77.4%) 1 signal	35 144 198 – 56 526 166 bp (65.2%)

^a^1 signal indicates the presence of the del(20)(q); ^b^a-CGH, array-based comparative genomic hybridization ^c^bp, base pairs; ^d^evaluated by the formula suggested by Valli et al. [13]; ^e^na, not available; ^f^patient with myelodysplastic syndrome (RCUD).

**Figure 1:**
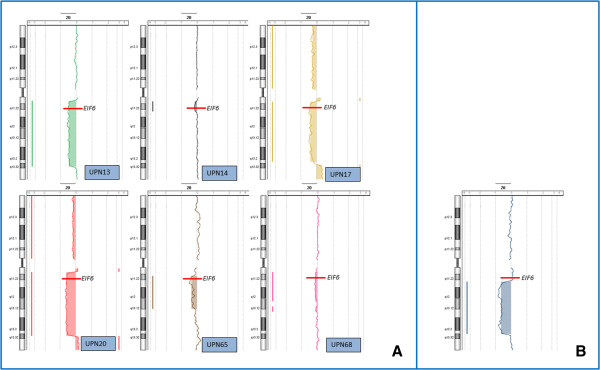
**a-CGH profiles of chromosome 20 in the 7 patients investigated. (A)** The six SDS patients, identified by their unique patient number (UPN), showing the extension of the interstitial deletion and the acquired loss of the *EIF6* gene; **(B)** Extension of the interstitial deletion in the RCUD patient: *EIF6* is not included in the deleted region.

The a-CGH results confirmed the interstitial deletion of the long arms of chromosome 20 in the six SDS patient and in the one affected by RCUD. They gave also evidence of the further rearrangements of the del(20) in subclones of the SDS patients UPN 17 and 20, but these changes superimposed to the initial one were already discussed [11] and are not object of the present report. The loss of material of all patients is illustrated in Figure 1, and the precise localization of the proximal and distal breakpoints leading to the deletion are given in Table 1. The difference among the six cases in shifting from the central line of the a-CGH profiles (Figure 1) is due to the different proportions of abnormal cells, which were known from chromosome and FISH analyses, but were also reassessed by calculating them from the a-CGH results themselves [13] (Table 1).

The a-CGH results concerning the region of chromosome 7 where the *SBDS* gene is located were normal in all patients, as expected.

## Discussion

We reviewed from the literature 102 patients affected by MDS (64 cases), AML (18), or MPN (20) with interstitial del(20)(q), in whom the deletion was analyzed by a-CGH or SNP-array [8,9,14-31]. We took into account only data concerning the long arms, and we excluded from the subsequent analysis of breakpoints ten cases with complex rearrangements and more than one region lost. In total, 92 cases were considered (56 MDS, 17 AML, 19 MPN): they showed interstitial deletions originated by breakpoints proximal to the centromere with base pair (bp) position ranging from 29.400 to 54.356 Mb, and distal ones with positions ranging from 34.338 to 62.966 Mb. The size of the material lost ranged from 0.054 to 30.260 Mb (average 18.153). All literature data taken into account are listed in the Additional file 1: Table S1. The cases with the smallest deletions (less than 500 Kb) are only five, and as the authors declare, they are not included in the Database of Genomic Variants [32], but no available data on parents exclude that they are in fact benign copy number variations (bCNV). We remark that the comparison of the a-CGH/SNP-array data with results of chromosome analyses is very difficult because in many cases the karyotype is very complex, with a number of ill-defined changes almost incredible: this comparison obviously may not be essential, but one should expect to have in most cases chromosome analyses more readable, so that the array data may better define chromosome anomalies already detected.

The data available definitely do not permit to establish a real and unique CDR. Some attempts have been made to identify one or two CDR in the literature: in some papers individual results of the single patients are given [8,15], in other ones a possible CDR is discussed without giving the individual data [33,34]. The CDRs so postulated are not in fact supported by most reported cases. The three smallest CDRs suggested by Milosevic et al. [34], e.g., with loss of material between bp positions 33.500 and 36.170 Mb, concern segments which are in fact lacking only in 52 out of the 92 cases that we collected from the literature: so, this evaluation seems not to be reliable. Only a rough conclusion is possible on this point: a segment around the bands 20q11.23-q12 represent only a more common region of deletion, with lacking segments of a size evaluable from 0.100 Mb [34] to 10.2 Mb [15].

The RCUD case here reported lacks a segment of 21.382 Mb with breakpoint positions at 35,144,198 and 56,526,166 bp (Table 1, Figure 1), that is an interstitial deletion quite similar to the ones more common in MDS. In our SDS patients the pattern of the loss of material is somehow more uniform than in MDS/AML/MPN, with some considerable differences. In three of the patients (UPN 13, 17, 20) the position of both the proximal and the distal breakpoints were in small clustered regions (Figure 1) of about 400 and 1800 Kb, respectively (Table 1). In these three cases the size of the material lost was 26.863, 24.688 and 25.957 Mb. In the other three patients (UPN 14, 65, 68) the proximal breakpoint was always in the same region, with an overall variability of 1100 Kb (Table 1); on the contrary the distal breakpoint was different: in UPN 14 the deletion was very small (4.146 Mb, with distal breakpoint at 35.309 Mb position) and had not been detected at chromosome analysis (Table 1); in UPN 65 the distal breakpoint was significantly different from UPN 13, 17 and 20, with the loss of a segment of 19.340 Mb (Figure 1, Table 1); in UPN 68 in fact two interstitial deletions were shown to be present, and a segment of 2.103 Mb was conserved between, being the more distal breakpoint at 47.373 Mb position (Figure 1, Table 1), with an overall loss of 14.007 Mb. So, the proximal breakpoint leading to the interstitial del(20)(q) in SDS is consistently in a position closer to the centromere than the vast majority of the del(20)(q) in MDS/AML/MPN. This implies in all SDS cases the loss of the *EIF6* gene, whereas this gene is lost only in 52 out of the 92 cases of MDS/AML/MPN that we took into account. Based on our a-CGH results on SDS patients, it is possible to identify a ~4 Mb CDR located between 31,294,381 and 35,309,353 bp positions: it includes more than fifty genes identified, and, up-to-date, the only gene of this region that could be associated with SDS molecular pathway is *EIF6*.

## Conclusion

The loss of chromosome 20 long arm material in MDS/AML/MPN is highly variable with no identifiable CDR, whereas in SDS the loss is more uniform: it seems to be often almost identical (3/6 among our patients), and this is probably also the case of most patients from the literature based on standard chromosome analyses, according to the morphology of the del(20)(q) which is available for some of the reported cases, and to the fact that it is probable that more subtle deletions could escape detection, as was the case of our patient UPN14. In some SDS patients the loss of material may be smaller, due to different distal breakpoints, but the proximal one remains in the same region, always closer to the centromere than the *EIF6* gene localization. We already postulated that the loss of *EIF6*, as consequence of the acquired del(20) in BM, plays a specific pathogenetic role with a lower risk of transformation into MDS/AML in SDS patients [5].

## Methods

Chromosome analyses were performed on BM with routine methods. FISH analyses were made on metaphases and on interphase nuclei by standard techniques with the following probes, informative for the deletion detected: RP11-17 F3 (UPN 13, 20, 65, and the RCUD patient), CTD-2550C9 (UPN 13), CTD-3092 L7 (UPN 14).

The a-CGH was performed with the 244 K genome-wide system (Agilent Technologies Inc., Santa Clara, CA, USA), according to the manufacturer’s instruction on DNA from BM sampled at the same dates of cytogenetic results summarized in Table 1. All map positions in the results refer to the genome assembly map hg19; as to literature data, we converted to map hg19 also the positions originally identified on the basis of preceding maps.

## Competing interests

The authors declare that they have no competing interests.

## Authors’ contributions

CM, BP, LM and GM contributed equally to chromosome analyses and FISH. RV and CM performed array-CGH analyses and mutational analysis of patients 1 and 2. FL, FP and EM conceived and coordinated the study, and drafted the manuscript. All authors have read and approved the final manuscript.

## Supplementary Material

Additional file 1: Table S1 Table with data on the position of proximal and distal breakpoints and on the size of material lost in the 92 cases of myelodysplastic syndrome (MDS), acute myeloid leukaemia (AML), and myeloid neoplasms (MPN) from the literature taken into account. Click here for file

## References

[B1] Dror Y Shwachman-Diamond syndrome Pediatr Blood Cancer 2005 6 892 901 10.1002/pbc.20478 16047374

[B2] Bilhou-Nabera C del(20q) in myeloid malignancies Atlas Genet Cytogenet Oncol Haematol 2000 http://AtlasGeneticsOncology.org/Anomalies/del20qID1040.html

[B3] Göhring G Karow A Steinemann D Wilkens L Lichter P Zeidler C Niemeyer C Welte K Schlegelberger B Chromosomal aberrations in congenital bone marrow failure disorders – an early indicator for leukemogenesis? Ann Hematol 2007 6 733 739 10.1007/s00277-007-0337-z 17653548

[B4] Liu JM A clinical algorithm predicts hematological complications in Shwachman-Diamond syndrome? Expert Rev Hematol 2012 6 373 375 10.1586/ehm.12.31 22992231

[B5] Pressato B Valli R Marletta C Mare L Montalbano G Lo Curto F Pasquali F Maserati E Deletion of chromosome 20 in bone marrow of patients with Shwachman-Diamond syndrome, loss of the *EIF6* gene and benign prognosis Br J Haematol 2012 6 503 505 10.1111/j.1365-2141.2012.09033.x 22295858

[B6] Wang PW Eisenbart JD Espinosa R III Davis EM Larson RA Le Beau MM Refinement of the smallest commonly deleted segment of chromosome 20 in malignant myeloid diseases and development of a PAC-based physical and transcription map Genomics 2000 6 28 39 10.1006/geno.2000.6215 10945467

[B7] Bench AJ Nacheva EP Hood TL Holden JL French L Swanton S Champion KM Li J Whittaker P Stavrides G Hunt AR Huntly BJ Campbell LJ Bentley DR Deloukas P Green AR together with the UK Cancer Cytogenetics Group (UKCCG) Chromosome 20 deletions in myeloid malignancies: reduction of the common deleted region, generation of a PAC/BAC contig and identification of candidate genes Oncogene 2000 6 3902 3913 10.1038/sj.onc.1203728 10952764

[B8] Huh J Tiu RV Gondek LP O’Keefe CL Jasek M Makishima H Jankowska AM Jiang Y Verma A Theil KS McDevitt MA Maciejewski JP Characterization of chromosome arm 20q abnormalities in myeloid malignancies using genome-wide single nucleotide polymorphism array analysis Genes Chromosomes Cancer 2010 6 390 399 20095039 10.1002/gcc.20748

[B9] Okada M Suto Y Hirai M Shiseki M Usami A Okajima K Teramura M Mori N Motoji T Microarray CGH analyses of chromosomal 20q deletions in patients with hematopoietic malignancies Cancer Genet 2012 6 18 24 10.1016/j.cancergen.2011.12.002 22429594

[B10] Maserati E Minelli A Pressato B Valli R Crescenzi B Stefanelli M Menna G Sainati L Poli F Panarello C Zecca M Lo Curto F Mecucci C Danesino C Pasquali F Shwachman syndrome as mutator phenotype responsible for myeloid dysplasia/neoplasia through karyotype instability and chromosome 7 and 20 anomalies Genes Chromosomes Cancer 2006 6 375 382 10.1002/gcc.20301 16382447

[B11] Maserati E Pressato B Valli R Minelli A Sainati L Patitucci F Marletta C Mastronuzzi A Poli F Lo Curto F Locatelli F Danesino C Pasquali F The route to development of myelodysplastic syndrome/acute myeloid leukaemia in Shwachman-Diamond syndrome: the role of ageing, karyotype instability, and acquired chromosome anomalies Br J Haematol 2009 6 190 197 10.1111/j.1365-2141.2009.07611.x 19222471

[B12] Valli R Marletta C Pressato B Montalbano G Lo Curto F Pasquali F Maserati E Comparative genomic hybridization on microarray (a-CGH) in constitutional and acquired mosaicism may detect as low as 8% abnormal cells Mol Cytogenet 2011 6 13 10.1186/1755-8166-4-13 21554683 PMC3101650

[B13] Valli R Maserati E Marletta C Pressato B Lo Curto F Pasquali F Evaluating chromosomal mosaicism by array comparative genomic hybridization in haematological malignancies: the proposal of a formula Cancer Genet 2011 6 216 218 10.1016/j.cancergen.2011.02.002 21536241

[B14] Gondek LP Tiu R O’Keefe CL Sekeres MA Theil KS Maciejevski JP Chromosomal lesions and uniparental disomy detected by SNP arrays in MDS, MDS/MPD, and MDS-derived AML Blood 2008 6 1534 1542 17954704 10.1182/blood-2007-05-092304 PMC2214746

[B15] Starczynowski DT Vercauteren S Telenius A Sung S Tohyama K Brooks-Wilson A Spinelli JJ Eaves CJ Eaves AC Horsman DE Lam WL Karsan AC High-resolution whole genome tiling path array CGH analysis of CD34^+^ cells from patients with low-risk myelodysplastic syndromes reveals cryptic copy number alterations and predicts overall and leukemia-free survival Blood 2008 6 3412 3424 10.1182/blood-2007-11-122028 18663149

[B16] Heinrichs S Kulkarmi RV Bueso-Ramos CE Levine RL Loh ML Li C Neuberg D Kornblau SM Issa J-P Gilliland DG Garcia-Manero G Kantarjian HM Estey EH Look AT Accurate detection of uniparental disomy and microdeletions by SNP array analysis in myelodysplastic syndromes with normal cytogenetics Leukemia 2009 6 1605 1613 10.1038/leu.2009.82 19387468 PMC2950785

[B17] Langemeijer SMC Kuiper RP Berends M Knops R Aslanyan MG Massop M Stevens-Linders E Van Hoogen P Geurts Van Kessel A Raymakers RAP Kamping EJ Verhoef GE Verburgh E Hagemeijer A Vendenberghe P De Witte T Van der Reijden BA Jansen JH Acquired mutations in *TET2* are common in myelodysplastic syndromes Nat Genet 2009 6 838 843 10.1038/ng.391 19483684

[B18] Borze I Juvonen E Ninomiya S Jee KJ Elonen E Knuutila S High-resolution oligonucleotide array comparative genomic hybridization study and methylation status of the *RPS14* gene in de novo myelodysplastic syndromes Cancer Genet Cytogenet 2010 6 166 173 10.1016/j.cancergencyto.2009.11.012 20193850

[B19] Slovak ML Smith DD Bedell V Hsu Y-H O’Donnell M Forman SJ Gaal K McDaniel L Schultz R Ballif BC Shaffer LG Assessing karyotype precision by microarray-based comparative genomic hybridization in the myelodysplastic/myeloproliferative syndromes Mol Cytogenet 2010 6 23 10.1186/1755-8166-3-23 21078186 PMC3000833

[B20] McKinnon RN Selan C Wall M Campbell LJ The paradox of 20q11.21 amplification in a subset of cases of myeloid malignancy with chromosome 20 deletion Genes Chromosomes Cancer 2010 6 998 1013 10.1002/gcc.20806 20645416

[B21] Barresi V Palumbo GA Musso N Consoli C Capizzi C Meli CR Romano A Di Raimondo F Condorelli DF Clonal selection of 11q CN-LOH and CBL gene mutation in a serially studied patient during MDS progression to AML Leuk Res 2010 6 1539 1542 10.1016/j.leukres.2010.07.004 20674974

[B22] Parkin B Erba H Ouillette P Roulston D Purkayastha A Karp J Talpaz M Kujawski L Shakhan S Li C Shedden K Malek SN Acquired genomic copy number aberrations and survival in adult acute myelogenous leukemia Blood 2010 6 4958 4967 10.1182/blood-2010-01-266999 20729466 PMC3012590

[B23] Praulich I Tauscher M Göhring G Glaser S Hofmann W Feurstein S Flotho C Lichter P Niemeyer CM Schlegelberger B Steinemann D Clonal heterogeneity in childhood myelodysplastic syndromes – challenge for the detection of chromosomal imbalances by array-CGH Genes Chromosomes Cancer 2010 6 885 900 10.1002/gcc.20797 20589934

[B24] McKinnon RN Kannourakis G Wall M Campbell LJ A cryptic deletion in 5q31.2 provides further evidence for a minimally deleted region in myelodysplastic syndromes Cancer Genet 2011 6 187 194 10.1016/j.cancergen.2011.02.001 21536236

[B25] Bajaj R Xu F Xiang B Wilcox K DiAdamo AJ Kumar R Pietraszkiewicz A Halene S Li P Evidence-based genomic diagnosis characterized chromosomal and cryptic imbalances in 30 elderly patients with myelodysplastic syndrome and acute myeloid leukemia Mol Cytogenet 2011 6 3 10.1186/1755-8166-4-3 21251322 PMC3031273

[B26] Klampfl T Harutyunyan A Berg T Gisslinger B Schalling M Bagienski K Olcaydu D Passamonti F Rimu E Pietra D Jäger R Pieri L Guglielmelli P Iacobucci I Martinelli G Cazzola M Vannucchi AM Gisslinger H Kralovics R Genome integrity of myeloproliferative neoplasms in chronic phase and during disease progression Blood 2011 6 167 176 10.1182/blood-2011-01-331678 21531982

[B27] Rice KL Lin X Wolniak K Ebert BL Berkofsky-Fessler W Buzzai M Sun Y Xi C Elkin P Levine R Golub T Gilliland DG Crispino JD Licht JD Zhang W Analysis of genomic aberrations and gene expression profiling identifies novel lesions and pathways in myeloproliferative neoplasms Blood Cancer J 2011 6 e40 10.1038/bcj.2011.39 22829077 PMC3256752

[B28] Kolquist KA Schultz RA Furrow A Brown TC Han J-Y Campbell LJ Wall M Slovak ML Shaffer LG Ballif BC Microarray-based comparative genomic hybridization of cancer targets reveals novel, recurrent genetic aberrations in the myelodysplastic syndromes Cancer Genet 2011 6 603 628 10.1016/j.cancergen.2011.10.004 22200086

[B29] Hahm C Mun YC Seong CM Chung WS Huh J Additional genomic aberrations identified by single nucleotide polymorphism array-based karyotyping in an acute myeloid leukemia case with isolated del(20q) abnormality Ann Lab Med 2012 6 445 449 10.3343/alm.2012.32.6.445 23130347 PMC3486942

[B30] Nowak D Klaumuenzer M Hanfstein B Mossner M Nolte F Nowak V Oblaender J Hecht A Hütter G Ogawa S Kohlmann A Haferlach C Schlegelberger B Braess J Seifarth W Fabarius A Erben P Saussele S Müller MC Reiter A Buechner T Weiss C Hofmann W-K Lengfelder E SNP array analysis of acute promyelocytic leukemia may be of prognostic relevance and identifies a potential high risk group with recurrent deletions on chromosomal subband 1q31.3 Genes Chromosomes Cancer 2012 6 756 767 10.1002/gcc.21961 22488577

[B31] Yi JH Huh J Kim H-J Kim S-H Kim SH Kim KH Do YR Mun Y-C Kim H Kim MK Kim H-J Kim T Kim DDH Genome-wide single-nucleotide polymorphism array-based karyotyping in myelodysplastic syndrome and chronic myelomonocytic leukemia and its impact on treatment outcomes following decitabine treatment Ann Hematol 2013 6 459 469 10.1007/s00277-012-1635-7 23262795

[B32] Database of genomic variants http://dgv.tcat.ca/

[B33] Schaub FX Jäger R Looser R Hao-Shen H Hermouet S Girodon F Tichelli A Gisslinger H Kralovics R Skoda RC Clonal analysis of deletions on chromosome 20q and *JAK2*-V617F in MPD suggests that del20q acts independently and is not one of the predisposing mutations for *JAK2*-V617F Blood 2009 6 2022 2027 10.1182/blood-2008-07-167056 19047681

[B34] Milosevic JD Puda A Malcovati L Berg T Hofbauer M Stukalov A Klampfl T Harutyunyan AS Gisslinger H Gisslinger B Burjanivova T Rumi E Pietra D Elena C Vannucchi AM Doubek M Dvorakova D Robesova B Wieser R Koller E Suvajdzic N Tomin D Tosic N Colinge J Racil Z Steurer M Pavlovic S Cazzola M Kralovics R Clinical significance of genetic aberrations in secondary acute myeloid leukemia Am J Hematol 2012 6 1010 1016 10.1002/ajh.23309 22887079

